# Molecular evolution accompanying functional divergence of duplicated genes along the plant starch biosynthesis pathway

**DOI:** 10.1186/1471-2148-14-103

**Published:** 2014-05-15

**Authors:** Odrade Nougué, Jonathan Corbi, Steven G Ball, Domenica Manicacci, Maud I Tenaillon

**Affiliations:** 1 Department of Evolutionary Ecology, CEFE-CNRS, UMR 5175, F-34293 Montpellier, Cedex 5, France; 2 Department of Ecology and Evolutionary Biology, U.C. Irvine, Irvine, California, USA; 3 University Paris-Sud, UMR 0320/UMR 8120 Génétique Végétale, Ferme du Moulon, F-91190 Gif-sur-Yvette, France; 4 Department of Plant Biology, University of Georgia, Athens, Georgia, USA; 5 Laboratoire de Glycobiologie Structurale et Fonctionnelle, UMR 8576, Université des Sciences et Technologies de Lille, F-59655 Villeneuve d’Ascq, Cedex, France; 6 CNRS, UMR 0320/UMR 8120 Génétique Végétale, Ferme du Moulon, F-91109 Gif-sur-Yvette, France

**Keywords:** Starch enzymes, Angiosperms, Positive selection, Paralogous genes, Escape from Adaptive Conflict model, Protein sequence evolution, PAML

## Abstract

**Background:**

Starch is the main source of carbon storage in the *Archaeplastida*. The starch biosynthesis pathway (sbp) emerged from cytosolic glycogen metabolism shortly after plastid endosymbiosis and was redirected to the plastid stroma during the green lineage divergence. The SBP is a complex network of genes, most of which are members of large multigene families. While some gene duplications occurred in the *Archaeplastida* ancestor, most were generated during the sbp redirection process, and the remaining few paralogs were generated through compartmentalization or tissue specialization during the evolution of the land plants. In the present study, we tested models of duplicated gene evolution in order to understand the evolutionary forces that have led to the development of SBP in angiosperms. We combined phylogenetic analyses and tests on the rates of evolution along branches emerging from major duplication events in six gene families encoding sbp enzymes.

**Results:**

We found evidence of positive selection along branches following cytosolic or plastidial specialization in two starch phosphorylases and identified numerous residues that exhibited changes in volume, polarity or charge. Starch synthases, branching and debranching enzymes functional specializations were also accompanied by accelerated evolution. However, none of the sites targeted by selection corresponded to known functional domains, catalytic or regulatory. Interestingly, among the 13 duplications tested, 7 exhibited evidence of positive selection in both branches emerging from the duplication, 2 in only one branch, and 4 in none of the branches.

**Conclusions:**

The majority of duplications were followed by accelerated evolution targeting specific residues along both branches. This pattern was consistent with the optimization of the two sub-functions originally fulfilled by the ancestral gene before duplication. Our results thereby provide strong support to the so-called “Escape from Adaptive Conflict” (EAC) model. Because none of the residues targeted by selection occurred in characterized functional domains, we propose that enzyme specialization has occurred through subtle changes in affinity, activity or interaction with other enzymes in complex formation, while the basic function defined by the catalytic domain has been maintained.

## Background

Living organisms store carbon as soluble (glycogen) or insoluble (starch) polysaccharides. Starch is a storage polysaccharide made of α-1.4-glucans with α-1.6 branches [1]. It is composed of two polymer fractions [2]: the moderately branched amylopectin, forming the semi-crystalline backbone of the starch granule [3,4]; and amylose, a fraction with very few branches, which is embedded inside the amylopectin matrix. The branching pattern of amylopectin is distinctively asymmetrical, allowing for the close packing of intertwined chains into helical structures that crystallise and collapse by dehydration, hence forming the starch granule [5,6].

Glycogen is widespread across archaea, eubacteria and eukaryotes [6,7]. In contrast, starch is found mostly in lineages derived from primary plastid endosymbiosis: the *Archaeplastida.* Starch is also occasionally found in some unicellular marine diazotrophic cyanobacteria and several secondary endosymbiotic lineages [8-10]. A majority of the enzymes in the starch biosynthesis pathway (sbp) are derived from members of the eukaryotic glycogen metabolism pathway. A few enzymes however display a prokaryotic phylogenetic affiliation. Among them, ADP-glucose pyrophosphorylase and Granule Bound Starch Synthase I were acquired through endosymbiotic gene transfer from the plastid ancestor. Additionally, isoamylases and soluble starch synthases III-IV were transmitted by lateral gene transfer from intracellular chlamydiae pathogens [9-11]. Finally archaeplastidal pullulanases are distinctively polyphyletic and were acquired from diverse unidentified proteobacterial sources.

It is generally acknowledged that the cyanobacterial and eukaryotic pathways of storage polysaccharide merged during plastid endosymbiosis to generate an ancient cytosolic starch biosynthesis pathway [12]. After or during metabolic transformation of the protoplastid into a true organelle, the *Archaeplastida* diverged into three lineages: the *Glaucophyta* (glaucophytes), the *Rhodophyceae* (red algae) and the *Chloroplastida* (green algae and land plants) [5,11]. While the ancient cytosolic localization of storage polysaccharides was maintained in red algae and glaucophytes, the green lineage redirected the whole sbp to the plastid stroma as it diverged from the other *Archaeplastida* lineages [13].

**Figure 1:**
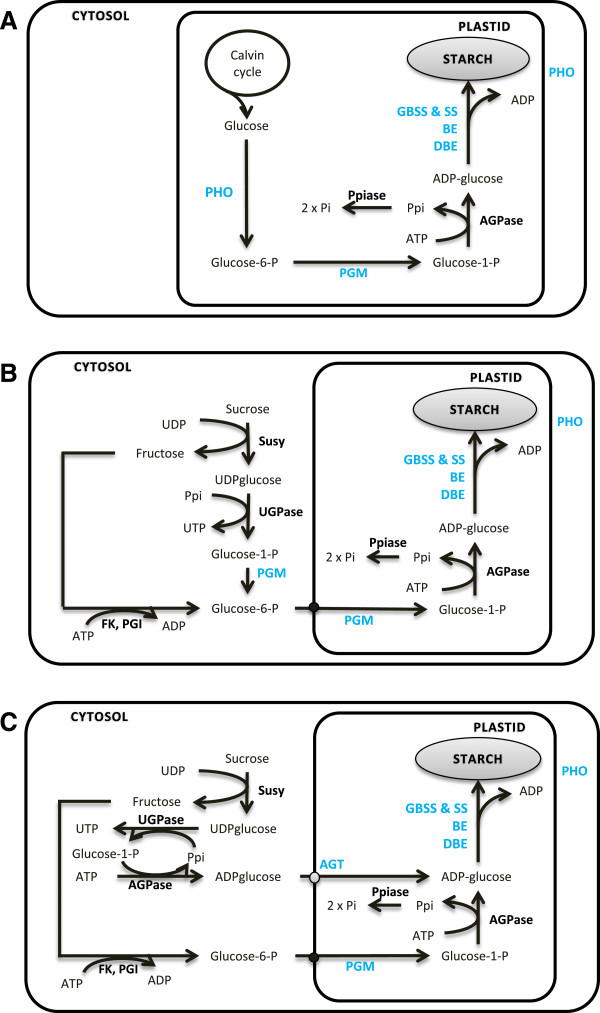
**Diversity of the starch biosynthesis pathway among organs(adapted from Comparot-Moss and Denyer, 2009 **[14]**).** Starch biosynthetic pathway in **A:** photosynthetic tissues; **B:** storage tissues and **C:** the *Poaceae* endosperm. Abbreviations for enzymes (blue or black, corresponding to enzymes included or not included in our study, respectively) are Susy, sucrose synthase; UGPase, UDP glucose pyrophosphorylase; PGM, phosphoglucomutase; FK, fructokinase; PGI, phosphoglucose isomerase; Ppiase, pyrophosphatase; AGPase, ADP glucose pyrophosphorylase; GBSS, granule bound starch synthase; SS, starch synthase; BE, starch-branching enzyme; DBE, debranching enzyme; PHO, phosphorylase.

Just as in green algae, in monocots and dicots the sbp involves a complex network of genes. However in monocots, ADP-glucose synthesis partitioning varies between source and storage tissues (Figure 1A and 1C; reviewed in [5,14,6]). Indeed ADP-glucose synthesis occurs both in the amyloplast and the cytosol of the cereal storage endosperm while it is otherwise confined to plastids in the leaves. Similarly the phosphoglucomutase enzyme (pgm) is present exclusively in the plastid of photosynthetic tissue but in both cytosol and plastid of storage tissue (Figure 1A and 1B). These pathways have been the basis of extensive physiological studies but few of them have explored the processes that govern their shaping. This issue is particularly relevant in the green lineage for which the sbp has been redirected to plastids and gene duplications have largely contributed to its organization and diversification.

Most genes involved in the sbp are members of multigenic families that have emerged from duplications during the complex *Archaeplastida* evolutionary history [15]. Gene duplications in the sbp have occurred at different times during *Archaeplastida* evolution. Some of them had already occurred in the *Archaeplastida* ancestor prior to divergence of *Chloroplastida* from *Rhodophyceae* and *Glaucophyta,* and possibly prior to plastid endosymbiosis, while others have occurred after the divergence of the three *Archaeplastida* lineages. Within *Chloroplastida*, redirection of the sbp from the cytosol to the evolving chloroplast was facilitated by gene duplications. Finally some duplications have occurred recently such as the duplication of Granule Bound Starch Synthase (gbss) within the *Poaceae* family.

Interestingly, Ball and Morell [16] reviewed the evolutionary history of duplications in three gene families involved in the sbp (starch synthase enzymes, branching enzymes and debranching enzymes), and have shown no functional redundancy among paralogs. Similarly, Yan *et al.*[17] reported 32 sbp genes in maize (*Zea mays*) and 27 in rice (*Oryza sativa*) and found that a substantial proportion of genes diverged in structure and/or expression pattern following whole genome duplications. Altogether, these results indicate that the sbp set up in plants is linked to the persistence of duplicated genes via functional specialization. The sbp set up therefore stands as an interesting framework to explore models of long-term persistence of duplicated genes and their contribution to pathway evolution.

Three models of persistence of duplicated genes are commonly encountered in the literature. The Duplication-Degeneration-Complementation (ddc) sub-functionalization model, first proposed by Force *et al.*[18], posits that the paralogs evolve complementary sub-functions, overall maintaining the ancestral function [19]. The Escape from Adaptive Conflict (eac) sub-functionalization model proposes the specialization of duplicated genes in two distinct functions originally fulfilled by the same ancestral gene [20,21]. Under this model, duplication resolves a conflict residing in the incapacity of improving simultaneously the two functions because of detrimental pleiotropic effects [19,22]. Finally, the neofunctionalization (neo-f) model [23] postulates that one paralog is recruited to fulfil a new function while the other preserves the ancestral function [22]. Variants of this last model have been proposed to resolve the so-called Ohno’s dilemma [24], *i.e.* loss of the neutrally evolving paralog before acquisition of a rare beneficial mutation [25]. For instance, the Innovation-Amplification-Divergence (iad) model [24] posits the evolution of a specialized enzyme from a progenitor enzyme displaying one or a range of promiscuous activities in addition to its primary function. These activities provide the substrate upon which natural selection can act and ultimately lead to functional divergence. In both the eac and iad models, several activities are assumed to exist prior to duplication. The specific resolution of Ohno’s dilemma by the iad model resides in the fact that changes in the ecological niche makes one or several pre-existing minor activities valuable. Selection will increase promiscuous activities, allowing maintenance of the paralog during its neo-functionalization [25].

These models differ in terms of selective pressures and molecular evolution rate following gene duplication [22], the latter being generally estimated using *ω*, the ratio of non-synonymous (*dN*) over synonymous (*dS*) substitution rates [26]. Hence, the ddc model predicts that the two paralogs evolve under selective neutrality (*ω* = 1) while accumulating complementary mutations leading to a loss of function. Under the eac model, both paralogs evolve under positive selection (*ω* > 1) allowing optimization of different sub-functions. In the neo-f and iad models, one paralog evolves under positive selection (*ω* > 1) as it is recruited for a new function or a previously neutral minor function, while the other paralog evolves under selective constraint (*ω* < 1) to preserve the ancestral function.

The neo-f model has been clearly illustrated through several examples of gain of function after duplication, such as the gain of an new enzyme function in glycosinolate synthesis in *Boechera*[27], the acquisition of the glutamate dehydrogenase gene in human and apes, that of the alcohol dehydrogenase gene in *Drosophila*, and those of gonadal paralogs of the pig cytochrome gene (for review see Conant and Wolfe [19]). iad illustrations come from the microbial literature, which offers several examples of new function evolution from the promiscuous activities of an ancestral enzyme (for a review see Soskine and Tawfik [28]). The distinction between the two neo-functionalization models, neo-f and iad, is challenging because it requires a knowledge of the promiscuous functions fulfilled by the ancestral gene – before duplication. Similarly, distinguishing between the two sub-functionalization models, ddc and eac, is difficult because both models rely on the partitioning of the ancestral function. So far, most of reported sub-functionalization cases have been interpreted in the light of the popular ddc model while the eac model has received little support in the literature. Des Marais and Rausher [21] identified clear evidence of eac from signs of adaptive evolution on two dihydroflavonol reductase paralogs in *Ipomea* and further suggest that evolution under the eac model may not be uncommon.

The fate of duplicated genes has been explored for one of the sbp enzymes among angiosperms, the ADP-Glucose Pyrophosphorylase (agp_ase_) [29,30]. In *Archaeplastida*, this protein is composed of two sub-units (one small and one large) encoded by paralogous genes originating from multiple duplication events. Patterns consistent with repeated sub-functionalization under the eac model have been described in the evolution of the agp_ase_ large sub-unit, leading to enzyme specialization for sink *versus* source tissues, as well as a particular agp_ase_ adaptation in grass endosperm [29]. Contrastingly, the sequence of the small sub-unit paralogs revealed evidence neither of sub-functionalization nor positive selection during angiosperms evolution, in spite of numerous duplication events. The small sub-unit has evolved under strong constraints, preventing the acquisition of new or modified functions [29,30]. Additionally, Corbi *et al.* revealed signs of coevolution among amino acid residues of the small sub-unit interaction domain [29] that likely also resulted from the strong evolutionary constraints placed on the agp_ase_ small sub-unit.

In the present study, we propose to extend the analysis of the evolutionary pattern following duplication events that occurred along the sbp evolution in angiosperms, to six gene families encoding sbp enzymes. We rely on phylogenetic approaches coupled with tests on the rates of evolution along branches and clades to assess selective processes that are responsible for the maintenance of paralogs along this pathway. More specifically, we compare observed patterns of evolution to those predicted by the ddc, the eac, and the neo-f/iad models. We discuss our results in the frame of the sbp evolution.

## Results

We studied the evolution of six gene families encoding enzymes of the sbp within angiosperms. For each family we identified paralogous genes maintained after duplication events that we matched to known compartmental or functional specialization. We further estimated the evolutionary rates in branches and clades emerging from such duplications to test whether accelerated evolution of paralogs has contributed to the evolution of this metabolic pathway. Patterns of evolutionary rates along branches were informative and provided support for distinct models of evolution of duplicated genes in the sbp pathway. In contrast, when performing pairwise comparisons of average *ω* values of clades emerging from gene duplication (data not shown), we found that all comparisons were significant (P-value < 7.35 10^-16^). Furthermore, *ω* values among clades varied between 0 and 0.674 consistent with purifying selection. Overall the clade model therefore did not detect positive selection and offered no power to discriminate among models. We therefore chose to focus primarily on the branch-site model in the presentation of our results.

### Paralogs with cytosolic vs. plastidic specialization

**Figure 2:**
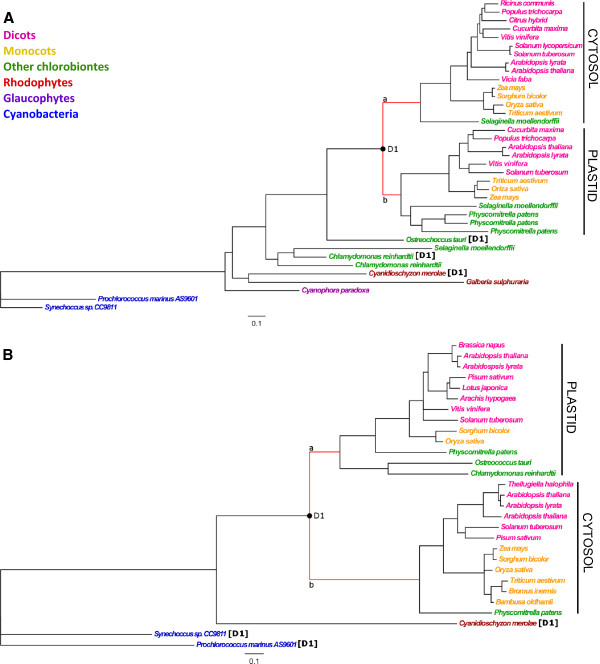
**Phylogenies of two starch biosynthesis enzymes exhibiting compartmental specialization. A:** Starch phosphorylase gene family (PHO); **B:** Phosphoglucomutase gene family (PGM). Paralogs are identified by species name. Phylogenies are rooted with *Prochlorococcus marinus* and *Synechoccus sp*. The scale is 0.1 substitutions per site. Nodes with bootstrap values lower than 90% were aggregated into rakes. Black circles indicate duplication events. Branches (a and b) emerging from duplications D1 were tested for deviation from neutral evolution. Corresponding outgroup sequences used to infer ancestral states are followed by duplication name (D1) in square brackets. Branches for which positive selection was detected are colored in red.

**Figure 3:**
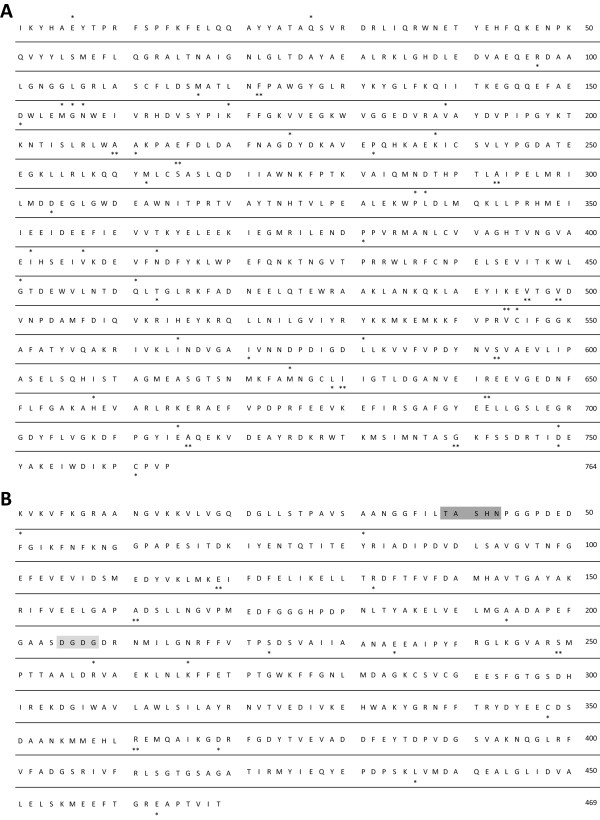
**Sites under selection along the evolution of the two starch biosynthesis enzymes exhibiting compartmental specialization, PHO and PGM. A:** Starch phosphorylase gene family; **B:** Phosphoglucomutase gene family. Sites are positioned on consensus sequences inferred at duplication nodes D1. Posterior probability (*PP*) of sites detected under positive selection in branch a (above the sequence) or b (beneath the sequence) are indicated: * for *PP >* 0.95, ** for *PP >* 0.99. Sites highlighted in dark grey and light grey correspond respectively to the catalytic domain and ion metal binding domain of pgm described by Manjunath and collaborators [30].

Both starch phosphorylase (Figure 2A; thereafter pho) and pgm (Figure 2B) phylogenies of gene families exhibited two groups of paralogs emerging from a duplication event (D1) that occurred before angiosperm radiation. Hence, each group of paralogs present sequences from *Selaginella moellendorffii* and/or *Physcomitrella patens* for pho and pgm gene families. Each group of paralogs displayed a cytosolic or plastidial expression specialization following D1 (Figure 2). We therefore tested for signatures of accelerated evolution along branches *a* and/or *b* emerging from duplication D1 in both pho and pgm.

pho exhibited positive selection and/or constraint relaxation in both branches D1a and D1b (Table I – pho). Evolution rate estimates revealed that about 15% and 6% sites– for branch D1a and branch D1b, respectively – evolved under positive selection. Using the beb (Bayes empirical Bayes) method we identified 26 and 22 sites in branches D1a and D1b respectively, with high posterior probability to have evolved under positive selection (Figure 3A).

Positive selection and constraint relaxation was observed in branches a and b for pgm (Table I – pgm). In branch D1a, about 9% of the sites were detected under positive selection with an *ω* value of 11.32 while in branch D1b, about 13% of the sites were detected under positive selection (*ω* = 7.37). beb revealed 4 and 12 sites under positive selection were identified in branches a and b, respectively (Figure 3B).

Among the 12 sites detected under positive selection in branch D1b (the branch leading to the cytosol-expressed paralog of the pgm enzyme), 4 of them (sites E_119,_ A_161_, S_249,_ R_361_) have a large posterior probability (above 0.99) to have evolved under positive selection. At position E_119_ (Figure 3B), a glutamic acid (E) was inferred in the ancestral sequence while, among plastidic paralogs, we found predominantly polar uncharged asparagine (N) or negatively charged aspartic (D) or glutamic (E) acids. In contrast, the paralogs expressed in the cytosol exhibited at this position a serine (S) or a threonine (T), two amino acids with polar uncharged side chains. At position A_161_ the ancestral sequence and the plastidic paralogs contained diverse residues with a predominance of proline (P) while only glutamic acid (E) was found in cytosolic paralogs. Residue S_249_ (Figure 3B) was a serine in all paralogs encoded by distinct codons, TCN for ancestral and plastidic sequences but AGY for cytosolic paralogs. Finally at R_361_, arginine (R) was the only residue found in the ancestral sequence and plastid-expressed paralogs, an amino acid with positively charged side chain, while the cytosolic paralogs carried two types of amino acids with hydrophobic side chains: valine (V) or isoleucine (I).

### Paralogs with functional specialization

In order to test if gene duplications in the evolutionary histories of the starch synthase, branching and debranching enzymes were accompanied by functional specialization following gene duplications, we tested for variation of evolutionary rates in branches emerging from these duplications.

### Starch synthase enzymes

**Figure 4:**
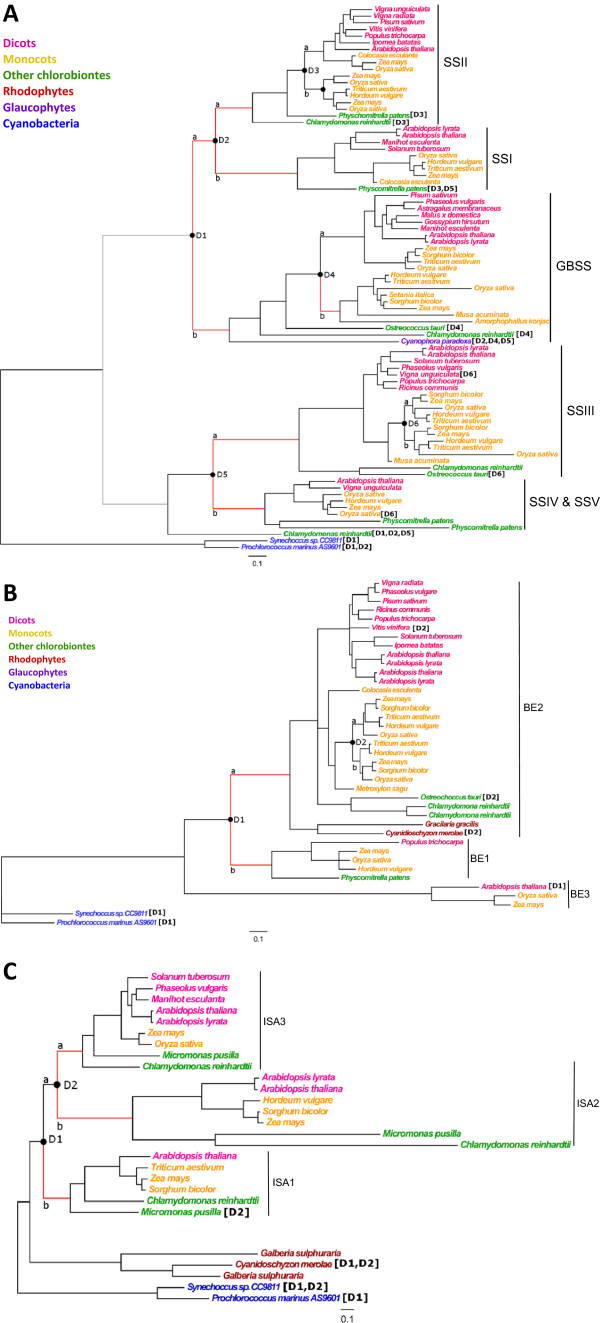
**Phylogenies of three starch biosynthesis enzymes exhibiting functional specialization. A:** Starch synthase gene family; **B:** Branching enzyme gene family; **C:** Debranching enzyme gene family. Paralogs are identified by species name. Phylogenies are rooted with *Prochlorococcus marinus* and *Synechoccus sp.* The scale is 0.1 substitutions per site. Nodes with bootstrap values lower than 90% were aggregated into rakes. Black circles indicate duplication events: 1 through n (n being 6 in A, 3 in B and 2 in C). Branches (a or b) emerging from duplications D1 through Dn were tested for deviation from non-neutral evolution. Corresponding outgroup sequences used to infer ancestral states are followed by duplication names in square brackets. Branches found to have evolved under positive selection are colored in red.

The phylogeny of the starch synthase family (Figure 4A) revealed three duplication events that occurred prior to the angiosperm radiation leading to the specialization in distinct functions. Duplications D1 and D2 led to three paralogous clades encoding gbss, ssi, and ssii enzymes, and duplication D5 led to clades ssiii, and ssiv&ssv. Each clade specialized in a distinct function in the emerging green lineage. Additional duplications (D3, D4 and D6) were observed subsequent to the angiosperm radiation. We tested whether an acceleration in evolution rate had occurred along branches emerging from duplications D1 to D6.

**Figure 5:**
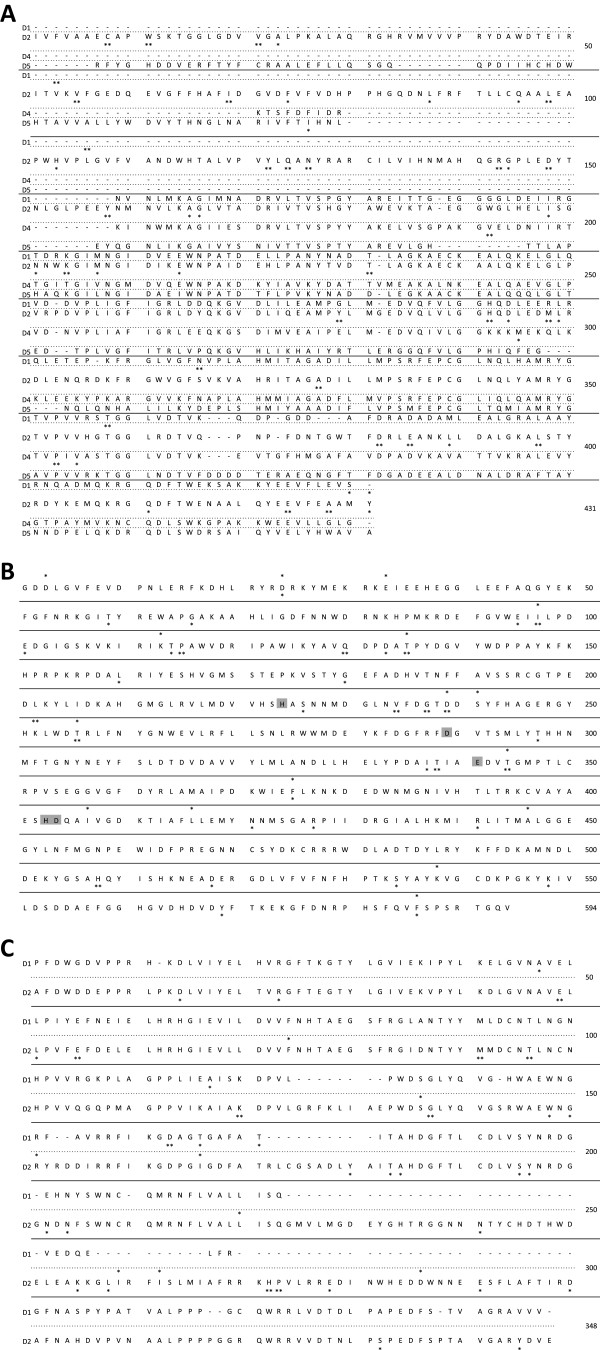
**Sites under selection along the evolution of the three starch biosynthesis enzymes exhibiting functional specialization, SS, BE and DBE. A:** Starch synthase gene family; **B:** Branching enzyme gene family (following D2); **C:** Debranching enzyme gene family. Sites are positioned on consensus sequences built for each gene family. Posterior probability (*PP*) of sites detected under positive selection in branch a (above the sequence) or b (beneath the sequence) are indicated: * for *PP >* 0.95, ** for *PP >* 0.99. Sites highlighted in dark grey in the be enzyme gene correspond to the catalytic domain described by Burton and collaborators [31].

Positive selection was observed along all branches following duplications D1, D2 and D5, prior to angiosperm radiation (Table I – gbss & ss). Approximately 20% and 35% of sites evolved under positive selection in branches D1a and D1b, respectively, while the beb method did not allow us to pinpoint any particular residue. In branches D2a and D2b, respectively, about 9% and 20% sites were detected under positive selection with *ω* estimated as 61.62 in branch D2a. Using the beb method, we were able to detect 6 and 40 sites under positive selection in D2a and D2b, respectively (Figure 5A). About 16% and 11% sites evolved under positive selection in branches D5a and D5b respectively, and the beb statistic allowed us to detect only one site as evolving under positive selection in branch D5b (Figure 5A).

For more recent duplications, posterior to angiosperm radiation, positive selection was detected only in branch D4b, with about 10% sites under positive selection and 4 sites detected using the beb method (Figure 5A). Note that the clade model revealed a higher *ω* value (0.286) in the clade emerging from the branch exhibiting accelerated evolution (D4b) than in the clade emerging from D4a (0.226).

### Branching enzymes

The branching enzyme (thereafter be) gene phylogeny presented three clades of paralogs, be1, be2 and be3 (Figure 4B). While the origin of the be3 clade remains unclear (see Discussion), be1 and be2 arose selectively in the *Chloroplastida* as they diverged from the other *Archaeplastida* and the pathway was redirected to the plastids (D1, Figure 4B). Two additional duplications, one specific to the *Poaceae* (D2) and one specific to the *Arabidopsis* genus, arose within be2 (Figure 4B). Positive selection or relaxation of constraint were detected in both branches D1a and D1b, with about 9% and 13% sites having evolved under positive selection, respectively (Table I – be). Using the beb method we were able to highlight 17 and 32 sites in branches D1a and D1b, respectively (Figure 5B). The structure of the branching enzyme family and the presence of five catalytic sites included in conserved domains has been well described in *Pisum sativum*[31], *Solanum tuberosum*[32], *Oryza sativa*[33] and *Sorghum bicolor*[34]. However, none of the sites that we detected under selection matched to these catalytic domains.

### Debranching enzymes

The debranching enzyme (thereafter dbe) gene phylogeny was composed of three clades of isoamylase genes (isa1, isa2 and isa3; Figure 4C) that arose from two duplication events (D1 and D2) prior to the angiosperm radiation. The evolutionary history of debranching enzyme genes is complex and has been very recently reviewed [35]. The duplications depicted here occurred as the pathway of the emerging green lineage was progressively reconstructed in plastids.

Acceleration in evolution rate was detected on the D1b branch, with about 9% sites under positive selection (Table I – dbe) and 5 sites were significant using the beb method (Figure 5C). Consistently with patterns observed for GBSS, the clade emerging from the branch displaying evidence of accelerated evolution also exhibited the greatest *ω* value (0.187 versus 0.143). About 9% and 23% of sites were detected under positive selection in branches D2a and D2b, respectively (Table I – dbe), and 8 and 28 sites detected with the beb method (Figure 5C).

### The Poaceae albumen-specific ADP-Glucose transporter

**Figure 6:**
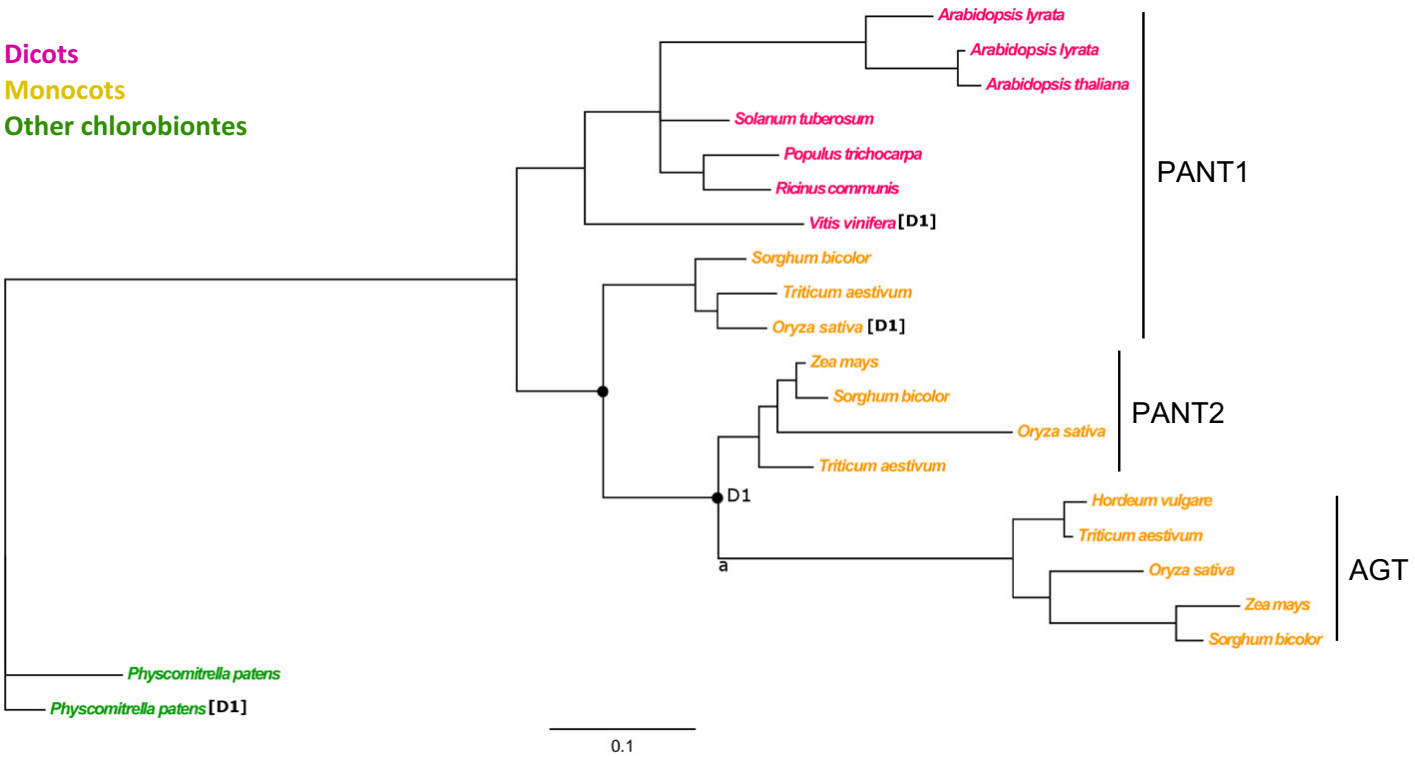
**Phylogeny of the ADP-Glucose transporter (AGT) and related transporters in plants, PANT1 and PANT2.** Paralogs are identified by species name. Phylogeny is rooted with *Physcomitrella patens*. The scale is 0.1 substitutions per site. Nodes with bootstrap values lower than 90% were aggregated into rakes. Black circles indicate duplication events. Branch a emerging from duplication D1 and leading to the agt clade was tested for deviation from non-neutral evolution. Corresponding outgroup sequences used to infer ancestral states are followed by duplication name in square brackets.

Genes coding for the ADP-glucose transporter (agt; Figure 6) are strictly restricted to *Poaceae*. We retrieved the sequences from related transporters in plants (pant1 and pant2) and built a phylogeny. The agt sequences form a monophyletic clade arising from pant2 through a duplication event (D1, Figure 6) prior to the *Poaceae* radiation. agt transports ADP–glucose through the plastid membrane, while pant proteins are known or assumed to be plastidial adenine nucleotide transporters [14], suggesting that the ancestral agt gene underwent neo-functionalization [23]. We thus tested for accelerated evolution in branch D1a that leads to the agt clade, but found no significant results (Table I – agt). Given the history of the transporter, this result is surprising and may result from reduced power, first because of the limited number of 10 aligned sequences. Second, the different transporters shared only 50% homology, thus the alignment was based only on the most conserved residues.

## Discussion

Upon gene duplication, loss is the expected fate of the majority of paralogs [36]. Evidence comes from the study of mutational effects of individual proteins [25]. For instance, Jacquier *et al.*[37] have shown that close to 50% of independent amino acid substitutions in a collection of 990 *Escherichia coli* mutants of the beta-lactamase TEM-1 exhibit deleterious effects as measured by a significant reduction of enzyme activity.

In this context, the starch biosynthetic pathway stands as an interesting example for studying the alternative fate, duplicated gene retention. Reconstruction of the sbp in the ancestor of *Archaeplastida* suggests that polysaccharide synthesis was ancestrally cytosolic, and then redirected to plastid at the origin of the *Chloroplastida*[11]. This change in protein addressing was clearly accompanied by numerous gene duplications leading to 32 and 27 genes involved in starch synthesis in maize and rice [17]. Interestingly, along the evolution of the *Chloroplastida*, complexification of the sbp was accompanied by an increase rate of paralog retention with fewer genes in *Chlorophyta* and *Bryophyta* (other chlorobiontes, Figures 2, 4, 6) as compared to angiosperms (Monocots and Dicots, Figures 2, 4, 6). This diversification is particularly prominent at the end of the synthesis pathway (for starch synthases, branching and debranching enzymes), where functions are fulfilled by a myriad of paralogs. The maintenance of so many paralogs has been possible because of the concomitant enzyme specialization and suggests that duplications were followed by sub-functionalization or neo-functionalization. In the present paper, we explore the evolutionary fate of genes from 6 families encoding sbp enzymes and revealed patterns of selection that have accompanied major gene duplication events and paralog preservation in the starch biosynthetic pathway.

### Cytosolic/Plastidic specialization

In the ancestor of *Archaeplastida*, as well as in extant glaucophytes and rhodophytes, the nucleotide-sugar substrates were used for chain elongation exclusively in the cytosol [11]. Multiple rounds of duplications for both pgm and pho genes have subsequently occurred during the green line radiation [13], one of which has given rise to a cytosolic/plastidic specialization (duplications D1 in Figures 2A and 2B). It is currently unknown where the ancestral genes of pgm or pho were expressed in chlorophytes [13]. We detected positive selection accompanying compartmental specialization for these two enzymes, i.e. along the two branches emerging from each duplication event (Figure 2). This pattern is compatible with the eac sub-functionalization model that assumes that both paralogs evolve under positive selection thereby improving two distinct functions or sub-functions initially fulfilled by the ancestral gene.

We detected numerous sites under positive selection in both pgm and pho. Two functional domains have been described in the pgm sequence: a catalytic domain and a metal ion binding domain [38]. The first site of the catalytic domain and the last site of the metal ion-binding domain differed between plastidic- and cytosolic-expressed paralog sequences but were not found to have evolved under positive selection (Figure 3B). Additionally, none of the pgm sites under positive selection occurred in these functional domains (Figure 3B). However, the sites we detected may be good candidates for future functional studies and help to reveal still unknown domains. Similarly, sites detected under positive selection in pho genes, for which no functional region is described, could help in the identification of crucial regions in this protein.

Along the branch that gave rise to the pgm cytosolic paralogs (D1b), positive selection was detected at 4 sites and was accompanied by changes in amino acid residues (Figure 3B). At position R_361_, amino acid residues with hydrophobic side chains replaced amino acid residues with positively charged side chains. This has likely modified the tertiary structure of the protein and its function and/or allosteric regulation since proteins are usually more stable with hydrophobic residues in the internal part of proteins (to avoid hydrophilic contact) while positively charged (polar) amino acids are mostly found on the protein surface.

At position S_249_, all paralogs shared the same amino acid encoded however by distinct codons, TCN for plastidic paralogs and ancestral sequence but AGY for cytosolic paralogs. Such a pattern of changes that involves multiple mutations could be explained by an initial deleterious mutation compensated by selection on subsequent mutations that restored the identity of the S residue.

### Functional specialization

Phylogenies of starch synthesis enzymes (ss, be and dbe) in the land plants reveal several duplications that occurred before angiosperm radiation (Figure 4) and led to a diverse panel of specialized enzymes that together insure the starch branching and debranching processes [9]. In the dbe family (Figure 4C), positive selection was observed in branch D1b but not D1a, suggesting that neo-functionalization accompanied the *Isa1* gene divergence. This result is in agreement with the existence of a distinct ancestral function for this enzyme. Indeed it is highly suspected that this enzyme emerged through duplication of a GlgX type of glucan hydrolase of chlamydial origin. In bacteria this enzyme displays a restricted substrate specificity in line with its function in glycogen catabolism. *Isa1* has evolved both a novel substrate specificity allowing it to debranch longer chains and also most probably a novel quaternary organisation into either the Isa1 dimer or the Isa1/Isa2 heteromultimer [32]. It is very likely that the ancestor of *Isa2* and *Isa3* genes maintained a GlgX-like function. Upon duplication *Isa2* acquired a function as a scaffolding subunit of the complex heteromultimeric Isa1/Isa2 enzyme. It lost its catalytic function in this process, which correlates with longer branches in maximum likelihood phylogenetic trees. *Isa3* on the other hand maintained some of GlgX restrictions with respect to substrate outer chain lengths but acquired the ability to accommodate debranching of a wider variety of branched oligosaccharides. Thus, the change in evolutionary rate we detected in both branches that gave rise to *Isa2* and *Isa3* (duplication D2) strongly suggest that positive selection rather constraint relaxation drove their divergence toward distinct specialized functions [35].

Multiple isoforms of starch synthase have been described in plants: the soluble forms ssi, ssii, ssiii, ssiv&ssv and the insoluble form gbss. Several authors studied the ss functional domains [7,39,40], and revealed a major catalytic domain of about 450 amino acids common to all starch synthases. This domain was aligned and used to build the phylogeny of starch synthases (Figure 4A). The ssi enzyme is involved in the synthesis of small chains of amylopectin. The ssii and ssiii play a major role in the synthesis of amylopectin, while the ssiv&ssv have a specific function in regulating the number of starch granules [2,16]. The ancestral function confined to amylose synthesis in the green lineage is still fulfilled by gbss. Duplications D1, D2 and D5 (Figure 4A) occurred during divergence of the *Chloroplastida* from the two other *Archaeplastida* (*Glaucophyta* and *Rhodophyceae*). We detected positive selection in branch D1a (Table 1) in accordance with the new functions fulfilled by ssi and ssii when they diverged from gbss. Detection of positive selection in branch D1b is more difficult to interpret. Following the gbss specific duplication D4, positive selection was detected in branch D4b but not D4a (Table 1), suggesting a pattern of neo-functionalization in the ancestral paralog of monocots. In this case neo-functionalization could very well comply with the iad model. Indeed gbssi in green algae was proven to exhibit two different modes of action for starch synthesis [41-44]. When the carbon flux to starch is low gbssi is chiefly concerned with the extension of amylopectin chains and the products of elongation remain embedded in the amylopectin structure. This situation is seen in green algae in mineral and nutrient supplied cultures where carbon is mostly directed to the cytosol to prepare for cell division [41]. The same situation is encountered in plant source tissues such as in leaves. When the flux to starch is very high as is the case in nutrient starved *Chlamydomonas reinhardtii*, gbssi synthesizes longer glucans which can be found free from the amylopectin chains [42,43]. This fraction is generally and classically defined as amylose [43]. It is possible that a duplication of the gbssi structural gene facilitated the selection of a gbssi more specialized in the high carbon flux mode, a function that pre-existed in all dicot and green algal gbssi enzymes. gbssi is highly expressed in endosperm of members of *Poaceae* and banana fruit pulp –high carbon flux organs – where it plays a critical role in starch accumulation [6,45].

**Table 1:** Detection of selection and/or relaxation of constraints by comparing MA, MA0 and M1a models (H1 hypothesis/H0 hypothesis)

**Gene family**	**D**^**a**^		**Seq.**^**b**^	**Length**^**c**^	**CF**^**d**^	**ω**_**b**_^**e**^	**Sites**^**f**^	**MA/M1a**^**g**^	**MA/MA0**^**g**^	**MA0/M1a**^**g**^
**PHO**	1	a	31	764	0	0.15	14.80	5.27 10^-16^	5.63 10^-7^	1.67 10^-11^
		b				0.15	5.70	1.65 10^-18^	8.88 10^-13^	2.83 10^-8^
**PGM**	1	a	29	469	0	0.11	8.88	6.55 10^-7^	3.92 10^-5^	6.72 10^-4^
		b				0.11	13.12	1.52 10^-12^	2.76 10^-6^	1.22 10^-8^
**GBSS&SS**	1	a	50	260	0	0.21	20.58	1.92 10^-7^	4.96 10^-6^	1.50 10^-3^
		b				0.22	34.86	2.70 10^-4^	8.32 10^-4^	n.s.
	2	a	29	425	0	0.19	8.91	9.47 10^-5^	1.73 10^-3^	n.s.
		b				0.18	19.18	1.19 10^-20^	3.11 10^-11^	5.09 10^-12^
	3	a	18	481	0	0.22	0.00	n.s.	n.s.	n.s.
		b				0.22	0.76	n.s.	n.s.	n.s.
	4	a	21	276	0	0.28	0.00	n.s.	n.s.	n.s.
		b				0.28	9.76	3.63 10^-6^	2.03 10^-5^	8.63 10^-3^
	5	a	30	324	0	0.20	16.39	6.01 10^-6^	3.24 10^-4^	8.54 10^-4^
		b				0.20	11.01	5.68 10^-7^	3.55 10^-6^	7.01 10^-3^
	6	a	13	426	1	0.25	0.00	n.s.	n.s.	n.s.
		b				0.25	0.00	n.s.	n.s.	n.s.
**BE**	1	a	35	594	0	0.14	9.07	1.40 10^-12^	2.62 10^-11^	1.44 10^-3^
		b				0.14	12.92	2.63 10^-19^	1.06 10^-12^	3.60 10^-9^
	2	a	13	674	1	0.15	26.61	n.s.	n.s.	n.s.
		b				0.15	0.43	n.s.	n.s.	n.s.
**DBE**	1	a	24	251	2	0.20	9.42	n.s.	n.s.	n.s.
		b				0.20	9.13	7.02 10^-5^	6.18 10^-4^	6.50 10^-3^
	2	a	18	348	0	0.17	9.94	8.71 10^-6^	5.21 10^-5^	8.48 10^-3^
		b				0.18	22.63	1.79 10^-9^	5.25 10^-5^	1.00 10^-6^
**AGT**	1	a	10	279	3	0.11	0.75	n.s.	n.s.	n.s.

^a^: duplication event D (Figures 2, 4 and 6) and the following branches a and b. ^b^: number of sequences. ^c^: number of sites. ^d^: codon substitution model. ^e^: *dN*/*dS* mean ratio value for background branches. ^f^: percentage of sites under selection in the forward branch. ^g^: LRT *p-values* for model comparisons. (*p-values* higher than 1% were considered non-significant, n.s.). Saturated branches (*dS >* 2.5) are not shown.

The branching enzymes be1 and be2 play different roles in the structure of amylopectin in storage organs [31,46]. The be1 knockout mutants observed display no particular phenotype, except in maize [4,16] where be1 appears to be required for starch mobilization during seed germination, and in *Chlamydomonas* where be1 mutants are defective for starch catabolism [7,47,48]. Additionally, the be1 paralog is absent from the *A. thaliana* genome [11], suggesting that it is not required for starch synthesis or mobilisation. In contrast, the be2 paralog has been more largely maintained, and plays a key role in starch synthesis [11,16]. Unexpectedly, we found evidence of positive selection in the branches giving rise to both be1 and be2 (Figure 4B), suggesting that the be1 paralog ancestor has initially evolved toward a specialized function that has secondarily been maintained in some taxa and lost in others. It is tempting to correlate this specialization to specific aspects of starch degradation during seed (for plants) or zygote (for green algae) germination.

Former results suggest that be3 is not directly issued from gene duplication but rather from an ancestral gene that could have pre-existed in the cytosolic glycogen metabolism network of the common ancestor of *Archaeplastida* before plastid endosymbiosis and which was lost from many *Archaeplastida*[11]. This may explain why the grouping of be3 with be1 and be2 is not well supported in our phylogeny (Figure 4B), making the study of selective pressures on the be3 ancestor irrelevant.

## Conclusions

We detected several instances of positive selection accompanying compartmentalization or functional specializations along the two branches emerging from duplication events at various steps during the evolution of the starch biosynthesis pathway. Several processes may generate these patterns including a combination of the above-cited models. For instance, sub-functionalization may be followed by independent improvements of functions but appeared as evidence for eac. We are also limited by the power of our analysis. Hence, positive selection may not be detected in a branch if the improvement of a pre-existing (major or promiscuous) function has been fulfilled by a single or very few mutations. Despite all these caveats, our study highlights a number of cases sustained by biological interpretations in favour of the eac sub-functionalization model. Our results thereby support its prominence along the evolutionary history of starch biosynthesis pathway.

In all multigene families studied here, none of the sites detected under positive selection matched with known functional domains of the proteins. At the angiosperm level, enzymes encoded by a given multigene family share the same basic function. For example, in the starch synthase enzyme family, all enzymes catalyse the same reaction that binds two glucoses in α-1,4 [16]. Differences between those enzymes are therefore subtle and have to do with the affinity for/production of amylopectin chains with distinct length and solubility. In the sbp, important interactions between enzymes exist. For example, Tetlow and collaborators [49] showed that in wheat, complex structures were formed through the association of be1, be2a and pho. Hence, complex formation and phosphorylation are required to activate be2a. Therefore, while the basic function (defined by the catalytic step) of every enzyme in a multigene family is constrained, function of the enzyme complex (defined by enzyme conformation and interaction with other enzymes during catalysis) may evolve after a duplication event. Our results suggest that new functions are generally acquired by mutations outside the highly conserved catalytic domains, most likely in regulatory domains and/or residues involved in changes in enzyme conformation/activity.

## Methods

### Sequence retrieval and alignment

We retrieved from the literature [11,12,14,17] the available coding sequences for six genes representative of six families (reference sequences) encoding starch biosynthesis enzymes: ADP-glucose transporter [agt: NM_119392.3, XM_002439325.1, XM_002438594.1], phosphoglucomutase [pgm: NM_001160993.1, AJ242601.1], debranching enzymes [dbe: NM_129551.3, NM_100213.3, NM_116971.5], branching enzymes [be: EF122471.1, NM_129196.3, AK118785.1], starch synthase [gbss&ss: AY149948.1, NM_122336.4, NM_110984.2, NM_101044.2, NM_117934.4] and starch phosphorylase [pho: NM_114564.2, AY049235.1]. In order to retrieve angiosperm sequences available for each gene family, we used all reference sequences as queries against the ncbi databases (http://www.ncbi.nlm.nih.gov/sites/gquery) using tBlastx. Sequences sharing more than 85% identity with any of the reference sequences were conserved, except for agt for which we used an identity threshold of 50% following [14]. In addition to angiosperm sequences, we also retrieved outgroup sequences from other chlorobiontes (*Chlamydomonas reinhardtii, Micromonas pusilla, Ostreococcus tauri, Physcomitrella patens, Selaginella moellendorffii*), rhodophytes (*Cyanidioschyzon merolae, Galdieria sulphuraria, Gracilaria gracilis*) and glaucophytes (*Cyanophora paradoxa*). Finally, we employed the same protocol using the BioCyc database (http://biocyc.org/) and a 60% identity threshold to retrieve cyanobacterial outgroup sequences from the *Prochlorococcus marinus* and the *Synechoccus* genome sequences.

In total, we retrieved 19, 23, 27, 31 and 23 angiosperm sequences plus 2, 7, 10, 8 and 14 outgroup (non-angiosperm) sequences for agt, pgm, dbe, be, gbss & ss and pho, respectively. The source and accession numbers of sequences analysed are indicated in Additional file 1. Protein sequence alignments were obtained using ClustalW in the bioedit 7.0.5.3 software (http://www.mbio.ncsu.edu/bioedit/bioedit.html; [50]), followed by manual inspection. Poorly aligned regions (>50% gap in local alignment) and insertion-deletions were excluded from alignments resulting in alignment lengths of 286, 285, 724, 628, 317 and 785 residues for agt, pgm, dbe, be, gbss & ss and pho respectively.

### Phylogenetic analysis

We used nucleotide sequences from the protein sequence alignments to build gene family phylogenies, except for agt and dbe for which we used protein alignments, due to greater divergence between sequences. We obtained phylogenies by Maximum Likelihood (ml) method using the phyml software (http://www.atgc-montpellier.fr/phyml/; [51]). We employed the gtr (General Time Reversible) substitution models determined by modeltest (https://code.google.com/p/jmodeltest2/; [52]). We rooted phylogenies with cyanobacteria as outgroups for all enzymes except AGT for which we used *Physcomitrella patens*. Bootstrap supports were calculated using 500 replicates.

### Detection of branches and residues deviating from neutral evolution

We checked our gene phylogenies with the known species phylogeny within each paralog [53]. No tree incongruence with the species evolution was observed except for few terminal branches whose nodes were poorly supported by low bootstrap values. In such cases, we left the nodes unresolved. Topologies used to test for evidence of deviation from neutral evolution along branches (names as a and b) emerging from major duplications (D) were based on these phylogenies.

We first determined for each phylogeny the most parsimonious equilibrium codon frequency model using codonfreq (CF) in the Site model M0 of codeml package (pamlv.4; [54]). We compared 4 nested models of codon frequency – equal frequencies (0 parameters – CF0), frequencies deduced from average nucleotide frequencies (3 parameters – CF1), frequencies deduced from average nucleotide frequencies at each codon position (9 parameters – CF2), frequencies different for each codon (60 parameters – CF3) – using likelihood ratio tests (LRTs). We retained the CF0 model for pgm, pho, be (D1 and D2) and gbss&ss (D1, D2, D3, D4 and D5), the CF1 model for be (D3) and gbss&ss (D6) and the CF3 model for dbe (D1 and D2) and agt.

Second, we used the output of the codon frequency model previously determined to compute *dS* for all branches of the phylogenies under the nearly neutral Site model (M1a) of the codeml package (pamlv.4; [54]). Note that all models we used in paml were named after [54]. This model allows the ratio *ω* to vary among sites [55]. Because models of sequence evolution rely on the infinite site model assumption, we discarded saturated branches, *i.e.* branches likely bearing sites with multiple substitutions, where *dS* value could not be estimated by paml.

Third, for the non-saturated target branches, we estimated the non-synonymous substitution rate (*dN*), the synonymous substitution rate (*dS*) and their ratio *ω* (*dN*/*dS*). In branch-site model A (MA), *ω* varies among sites and branches thereby allowing to estimate the proportion of sites subject to contrasted evolution rate along target branches (foreground branches) and background branches [56]. These models were compared using lrts as described by Yang [57]. Significance between Branch-Site model A (MA) and the null Branch-Site model A (MA0) reveals signs of positive selection, while significant differences between MA and the nearly neutral site model (M1a) can be interpreted as evidence for either relaxation of constraint and/or positive selection. Finally, significant LRT comparing MA0 to M1a indicates relaxed constraints [58].

When the lrt was significant (using a 0.01 α threshold), the beb (Bayes empirical Bayes) method was used to identify residues that are likely to have evolved under positive selection [56], based on a posterior probability threshold of 0.95. Consensus sequences were implemented simultaneously by paml, at each node of the phylogenies. We used each of the reconstructed ancestral sequences at target nodes to position residues.

In order to test for the long-term effect of selection after gene duplication, we used the Clade model C [59] that aims at detecting a difference in the rates of evolution between both clades emerging from target duplications. This model allows estimating the proportion of sites evolving at different *ω* rates in both clades, and is tested against model M1a using an LRT with 3 df.

### Availability of supporting data

All alignments and phylogenies used in the present paper are available on the Dryad repository at doi:http://10.5061/dryad.vj7nr.

## Abbreviations

sbp: starch biosynthesis pathway; susy: sucrose synthase; ugpase: UDP glucose pyrophosphorylase; fk: fructokinase; pgi: phosphoglucose isomerase; ppiase: pyrophosphatase; agt: ADP-glucose transporter; pgm: phosphoglucomutase; dbe: debranching enzymes; be: branching enzymes; gbss & ss: granule bound starch synthase and starch synthase; pho: starch phosphorylase; ddc: Duplication-Degeneration-Complementation; eac: Escape from Adaptive Conflict; iad: Innovation-Amplification-Divergence; neo-f: neofunctionalization.

## Competing interests

The authors declare that they have no competing interests.

## Authors’ contributions

ON carried out the sequences retrieval and data analyses, and wrote the first version of the manuscript. JC contributed to PAML analysis and data interpretation. SB contributed to results discussion and brought insightful comments on the manuscript. MIT and DM conceived the study, helped in data analysis and manuscript writing. All authors read and approved the final manuscript.

## Supplementary Material

Additional file 1 **List of sequences used in this study.** Each page of the excel file correspond to a gene family. For each sequence, we indicated the “Accession number”, the “Species name” and the “Gene name” when known. “References” indicates the source of the reference sequences used as queries in BLAST searches. “Sources” refers to screened databases. Click here for file
